# Ethical challenges and moral conflicts in periviability prenatal counseling: a scoping review of healthcare professionals’ perspectives

**DOI:** 10.1186/s12910-026-01464-w

**Published:** 2026-05-12

**Authors:** Alessia Bonaccorso, Antonella Nespoli, Elena Ferioli, Mario Picozzi

**Affiliations:** 1 Midwifery Education Program, University of Insubria, Via Ottorino Rossi 9, Varese, 21100 Italy; 2 Research Centre in Clinical Ethics (CREC), Department of Biotechnology and Life Sciences, University of Insubria, Varese, Italy; 3 School of Medicine and Surgery, University of Milano-Bicocca, Monza, Italy

**Keywords:** Periviability, Prenatal Counselling, Clinical Ethics Consultation, Ethical Issues, Moral Conflicts, Healthcare Professionals, Maternal–Fetal Medicine, Neonatal Intensive Care

## Abstract

**Background:**

Prenatal counselling at the threshold of viability raises profound ethical challenges for healthcare professionals. Decisions occur under conditions of prognostic uncertainty, high emotional intensity, and variable institutional norms. This scoping review aimed to map the ethical implications and moral conflicts perceived by healthcare professionals involved in periviability counselling, including how counselling is conducted, which contextual factors shape decision-making, and the perceived role and value of Clinical Ethics Consultation (CEC).

**Methods:**

The review followed the PRISMA-ScR guidelines and the Joanna Briggs Institute framework (JBI), using a PCC structure. A comprehensive search was conducted in PubMed, CINAHL, APA PsycInfo, Psychology and Behavioral Sciences Collection and Web of Science. Eligible studies included qualitative, quantitative, or mixed-methods research published in English and reporting on ethical issues, moral dilemmas, or decision-making among healthcare professionals involved in prenatal or periviability counselling. Editorials, commentaries, conference abstracts, theoretical papers, and studies focused solely on parental perspectives were excluded. Two reviewers independently screened all records using CADIMA, with disagreements resolved by consensus with a third reviewer. Data were charted and synthesized through descriptive numerical analysis and inductive thematic synthesis.

**Results:**

Twenty-five studies met inclusion criteria: 14 surveys, 9 qualitative studies, 1 mixed-methods study, and 1 randomized controlled trial (RCT). Healthcare professionals represented included neonatologists, obstetric and maternal–fetal medicine clinicians, nurses, trainees and ethicists. Six major themes emerged across study types: (1) variability in resuscitation thresholds and counselling practices; (2) pervasive prognostic uncertainty; (3) value pluralism and interprofessional divergence; (4) communication challenges in high-stakes encounters; (5) institutional culture and system-level influences; (6) moral distress and emotional burden among professionals. CEC was rarely integrated into practice: 17 studies did not mention it, 7 discussed it only conceptually, and only 1 described its operational use.

**Conclusions:**

Periviability counselling is ethically complex and inconsistently implemented across settings. Professionals navigate uncertainty, divergent values, and institutional pressures with limited structured support, while appropriate shared decision-making remains inconsistently achieved. Although CEC holds considerable potential value, it is still rarely used. Strengthening counselling practice will require clearer frameworks, improved communication strategies, interprofessional collaboration and the meaningful integration of ethical support systems.

**Supplementary Information:**

The online version contains supplementary material available at 10.1186/s12910-026-01464-w.

## Key message

• Counselling at the threshold of viability is ethically complex and variably structured across settings.
• Prognostic uncertainty and divergent professional roles complicate communication with families.
• Parental values and best-interest considerations are both central but often difficult to reconcile.
• Shared decision-making is widely endorsed yet inconsistently implemented in practice.
• Moral distress is common—especially among nurses and junior staff—and often insufficiently addressed.
• Clinical Ethics Consultation may support deliberation in ethically complex situations but remains rarely integrated or empirically evaluated.
• Institutional culture and policy strongly shape counselling pathways and decision thresholds.
• Professional perspectives beyond neonatology (OB/MFM, midwifery, nursing, ethics) remain underrepresented in both practice and research.

**Supplementary Information:**

The online version contains supplementary material available at 10.1186/s12910-026-01464-w.

## Background

Periviability refers to the period surrounding the threshold of fetal viability, conventionally located between approximately 22 and 25 weeks of gestation, during which neonatal survival remains highly uncertain and strongly dependent on centre-level practices and proactive care. Some professional bodies, such as the American College of Obstetricians and Gynecologists, extend the concept of periviability to approximately 20–25 weeks of gestation, reflecting an obstetric perspective on the earliest limits of potential survival [1]. In clinical neonatology and counselling practice, however, active decision-making most commonly focuses on the narrower range of 22–25 weeks, where survival becomes technically possible but remains highly uncertain.

This gestational boundary is not fixed and has evolved over time alongside advances in neonatal care. In high-resource settings, improvements in technology and clinical expertise have progressively lowered the gestational age at which survival may be possible, whereas in other regions viability thresholds remain higher due to differences in infrastructure, resources, and clinical practice. Viability should therefore be understood as a historically dynamic and globally variable construct rather than a uniform biological limit.

Recent population-based and meta-analytic data show substantial variation in survival for infants born at 22–25 weeks across regions and health systems, with survival at 22 weeks remaining low and increasing progressively with each additional week of gestation [2–4].

Although these births account for a small proportion of all deliveries—approximately 0.4% of births in the United States in 2015—they contribute disproportionately to neonatal mortality and serious disability, representing around 40% of neonatal deaths [5]. Recent advances in perinatal and neonatal intensive care have shifted survival curves, making active treatment at 22–23 weeks technically possible in some centres, yet with marked variability in policies, resource allocation and outcomes across countries and within health systems [2, 6]. These differences are further amplified at a global level. Rates of prematurity remain highest in many low- and middle-income countries, where limited access to specialised neonatal care, workforce shortages, and resource constraints contribute to substantially higher mortality and morbidity among extremely preterm infants [7]. Such structural inequalities not only affect clinical outcomes but also shape the ethical context in which counselling and treatment decisions are made, underscoring the importance of interpreting periviability within broader global health disparities.

Periviability has thus been described as a phase of “technologically assisted potential survival”, in which the possibility of survival is created and sustained almost entirely by invasive, resource-intensive interventions, while prognostic uncertainty remains high [8].

The ethical landscape at the limit of viability is shaped by the tension between improving survival and the persistent risk of serious impairment, by wide international variation in thresholds for initiating active care, and by differences in the availability and allocation of healthcare resources, which may influence decisions in settings where intensive neonatal care is resource-intensive or constrained. While in some settings resuscitation is routinely offered from 22 weeks, others recommend comfort care up to 24 weeks or adopt more flexible “grey zone” frameworks, where gestational age (GA) is only one among several prognostic factors [2, 3, 6, 9, 10]. Bioethical and professional guidance has increasingly criticised rigid gestational-age cut-offs, arguing that they inadequately reflect prognostic complexity and may be unjust to extremely preterm infants by denying potentially beneficial treatment purely based on estimated GA [1, 9, 11]. In Italy, the Italian National Bioethics Committee and the Rome Charter Guidelines have recommended moving away from absolute chronological thresholds towards an integrated assessment that considers prognostic uncertainty, limits to therapeutic obstinacy, and the interests of the newborn in dialogue with the parents [12, 13].

Antenatal consultations typically involve obstetricians, neonatologists, midwives, nurses and, in some settings, clinical ethicists, who are tasked with presenting possible scenarios, discussing options for care after birth—including the potential for intensive treatment, comfort-focused care, or intermediate approaches—and supporting parents in shared decision-making (SDM) [14–16]. In this review, the term *counselling* refers to the communicative and decision-support processes through which healthcare professionals provide information, discuss prognostic uncertainty, and explore treatment options with parents in situations of periviability. This use of the term is distinct from *ethics consultation*, which denotes a formal process of ethical analysis and support provided by clinical ethicists or ethics committees.

A recent scoping review of prenatal counseling for extreme prematurity at the limit of viability has highlighted personalization, parental values, uncertainty, emotions and the quality of the parent–physician relationship as central features of current practice [17]. At the same time, viewpoint papers have underscored the limitations of probabilistic evidence and outcome statistics in real-world counseling encounters, arguing that attempts to provide ever more detailed risk figures may overwhelm parents and fail to capture the affective and existential dimensions of decision-making [18]. These authors advocate for a more individualized, narrative-based approach that attends to what parents want and need, rather than treating counseling as the mere transmission of numerical information. Recent work on uncertainty and prognostic communication similarly emphasizes structured yet flexible frameworks that openly acknowledge prognostic uncertainty while supporting parents’ hopes and information needs [19, 20].

Despite these developments, studies conducted mainly in high-income settings continue to report persisting heterogeneity in counselling content, style and goals across and within countries. Professional societies such as the American Academy of Pediatrics have recommended multidisciplinary, culturally sensitive counseling that is honest about prognostic limits and explicitly oriented towards shared decision-making, but empirical studies show that parents often perceive inconsistent messages, and clinicians themselves report uncertainty about how to balance transparency, empathy and realism [21–25]. Moreover, professional guidelines and policy statements — including those issued by international bodies on periviability and neonatal decision-making [1, 11], and, in Italy, by the National Bioethics Committee and the Rome Charter Guidelines [12, 13] — focus primarily on what ought to be done, specifying who should decide, on what basis, and with which ethical justification, while devoting less attention to how healthcare professionals actually experience and navigate the moral complexity of these encounters in practice. An increasing number of studies have documented substantial emotional burden and moral distress among healthcare professionals involved in periviable care and neonatal intensive care, including during counselling and decision-making processes. Clinicians describe feelings of powerlessness, conflict of conscience, and distress when they perceive a gap between what they believe would be in the best interests of the infant and the course of action that is pursued, whether because of parental wishes, institutional pressures or divergent views within the team [26–29]. Recent studies — including systematic syntheses of moral distress in neonatal care [30] and qualitative research involving neonatologists and perinatal clinicians [31] — suggest that such distress often emerges not only from bedside care itself but also from the ethical tensions inherent in counselling families under conditions of uncertainty and value pluralism, contributing to emotional exhaustion and impaired professional wellbeing [32, 33]. Over time, moral distress and “moral residue” may accumulate, increasing the risk of burnout and erosion of moral integrity if not adequately recognized and addressed [34, 35]. Classic and more recent work in neonatal ethics has further highlighted how disagreements about limiting or withdrawing intensive care, differing conceptions of quality of life, and uncertainties about the infant’s moral status at the threshold of viability generate recurring ethical tensions that often become visible during counselling encounters and deliberative processes [10, 36].

CEC has been proposed as a potentially valuable form of ethics support in perinatal and neonatal care, particularly in decision-making at the limit of viability. CEC aims to provide a structured process for analysing ethically complex cases, clarifying values and obligations, mediating conflicts, and facilitating communication between professionals and families [37–39]. In perinatal and neonatal care, ethics consultation may help teams articulate the moral justification for initiating, continuing or limiting intensive treatment at the limit of viability, and support shared decision-making that is both ethically defensible and psychologically sustainable for those involved [40, 41]. Recent studies have likewise started to examine how ethics consultation services are used and valued in Neonatal Intensive Care Units (NICUs), but current evidence is limited to particular national settings and indicates considerable variability in practice [42]. Overall, the actual frequency, timing, indications and perceived impact of CEC in the specific context of prenatal counseling for periviable birth remain poorly documented. Existing reviews of ethics consultation tend to aggregate diverse clinical areas and rarely focus on obstetric and neonatal cases, while periviability-focused ethical reviews often discuss ethical principles and policy implications without examining how frontline professionals experience and evaluate ethics support in their daily practice [10, 38].

Despite extensive ethical, clinical, and policy debates, important knowledge gaps persist regarding how healthcare professionals experience the ethical complexity of periviable prenatal counseling, the types of moral conflicts they face, and how they perceive the role of CEC in these situations.

Given the conceptual complexity and methodological heterogeneity of the literature addressing ethical issues in periviability—ranging from qualitative studies on clinicians’ lived experiences, to surveys of attitudes, to ethical analyses and policy documents—a scoping review approach is particularly appropriate. Scoping reviews are especially suited to mapping broad and diverse bodies of evidence, clarifying how key concepts have been defined and applied across studies, and identifying gaps that require further empirical investigation. This methodological choice allows us to synthesise what is known about healthcare professionals’ moral experiences in prenatal counseling at the limits of viability, the types of ethical and moral conflicts reported, and the role and perceived impact of CEC within these contexts.

This scoping review aims to explore and map the existing literature on the ethical implications and moral conflicts experienced by healthcare professionals (Population) involved in prenatal counselling for anticipated periviable birth (Context**)**. It examines how ethical issues, moral distress, decision-making challenges and counselling practices are described (Concept), and explores the perceived role, use and value of CEC within Maternal–Fetal Medicine (MFM) and Neonatal Intensive Care Units (NICUs). The review also seeks to identify how contextual or institutional factors shape ethical decision-making in periviability counselling, and to highlight gaps that may inform future empirical and normative research.

## Methods

### Scoping review protocol and registration

This scoping review was conducted in accordance with the methodology proposed by the JBI [43] and the PRISMA-ScR guidelines (Preferred Reporting Items for Systematic Reviews and Meta-Analyses extension for Scoping Reviews) [44].

The choice of a scoping review was motivated by the exploratory nature of the research question: the aim was to systematically and transparently map the existing literature, identify knowledge gaps, and synthesise emerging evidence, rather than assess the effectiveness of specific interventions.

An a priori protocol was developed, registered, and disseminated publicly via the Open Science Framework (OSF) (https://osf.io/4zqgj/). Registration ensured transparency and traceability of the methodological process. The primary and secondary research questions, developed according to the PCC framework, are reported in the OSF protocol and summarised in the Introduction.

### Eligibility criteria

The eligibility criteria were defined according to the PCC (Population, Concept, Context) framework recommended by the JBI, to ensure methodological coherence and the relevance of selected studies to the objectives of the review. Studies were considered eligible if they involved healthcare professionals engaged in prenatal counselling in periviability contexts, including obstetricians, neonatologists, midwives, nurses, and clinical ethicists. The review focused on the ethical and moral implications experienced or perceived by these professionals, with particular attention to the presence of moral conflicts and the possible use of CEC. Our clinical focus was prenatal counselling for pregnancies between 22 and 25 weeks’ gestation, usually delivered in maternal–fetal medicine units or neonatal intensive care settings. Eligible sources included primary research adopting qualitative, quantitative, or mixed-methods designs, as well as pertinent systematic reviews and grey literature documents produced by institutional bodies or recognised scientific societies. To ensure the contemporaneity of the evidence and its alignment with recent developments in clinical practice and ethical discourse on periviability, temporal limits were applied, restricting inclusion to publications from January 2015 to July 2025. Only studies published in English were considered, both to ensure accessibility and to maintain adequate standards of methodological quality and reporting. Non-empirical contributions—such as editorials, commentaries, letters, and theoretical papers—were excluded, as were studies focusing exclusively on parental perspectives, conference abstracts without full text, and publications in languages other than English. Regarding grey literature, inclusion was limited to documents deemed methodologically transparent and originating from accredited organisations, to ensure their reliability and relevance. Studies were included only when ethical issues were explicitly explored as a primary analytical focus—such as moral dilemmas, ethical conflicts, moral distress, or value-based decision-making processes—rather than remaining implicit within broader clinical or policy discussions. Accordingly, surveys addressing thresholds of care, resuscitation practices, or guideline preferences were excluded when they did not substantively analyse the ethical dimensions of these decisions.

### Information sources

The search strategy was designed to systematically and comprehensively identify literature relevant to the themes addressed in the review. The process incorporated three main conceptual areas corresponding to the PCC framework: (1) periviability; (2) the ethical and moral dimensions associated with prenatal counselling and decision-making; and (3) the healthcare professionals involved in these processes. In this review, we use the expression *ethical* and *moral dimensions* as an operational umbrella term to refer to value-laden issues such as dilemmas, conflicts, and forms of moral distress in clinical practice, without assuming a specific philosophical distinction between ethics and morality.

The literature search was conducted in the databases PubMed/MEDLINE, CINAHL, APA PsycINFO, Web of Science, and Psychology and Behavioral Sciences Collection, selected for their complementary disciplinary coverage and their relevance to health sciences, clinical psychology, and bioethics. Search strategies were adapted to each database using a combination of controlled vocabularies—such as MeSH, CINAHL Headings, and the APA Thesaurus—and free-text terms, with the aim of maximising both sensitivity and specificity.

Temporal limits were applied to include publications from January 2015 to July 2025, and only English-language sources were considered. The most recent search was conducted in July 2025. To enhance the comprehensiveness of the search, the electronic database search was supplemented by manual screening of the reference lists of included studies and by consulting guidelines, recommendations, and institutional documents issued by national and international scientific societies and professional bodies in the fields of neonatology, clinical ethics, and maternal–fetal medicine.

### Search

The electronic search was designed and conducted to ensure maximum reproducibility, in accordance with PRISMA-ScR recommendations. The complete search strategy is reported for PubMed/MEDLINE, selected as a representative example of the approach used across all databases. For PubMed, the search combined free-text terms and controlled vocabulary (MeSH) structured around the three conceptual domains of the PCC framework: periviability, ethical and moral dimensions, and healthcare professionals involved in prenatal counselling. The full PubMed/MEDLINE search string was as follows:

- ((“Perinatal Care“[Mesh] OR “Fetal Viability“[Mesh] OR periviable[tiab] OR “peri-viability“[tiab] OR periviability[tiab] OR “limit of viability“[tiab] OR “fetal viability“[tiab] OR “periviable birth“[tiab] OR “peri-viability birth“[tiab] OR “peri-viable birth“[tiab])
- AND
- (“Bioethical Issues“[Mesh] OR “Ethics“[Mesh] OR “Ethics Consultation“[Mesh] OR bioethics[tiab] OR “ethical issues“[tiab] OR ethics[tiab] OR “ethical dilemma“[tiab] OR “ethical concerns“[tiab])
- AND
- ("Health Personnel"[Mesh] OR "Health Occupations"[Mesh] OR healthcare workers[tiab] OR healthcare professionals[tiab] OR healthcare providers[tiab] OR healthcare personnel[tiab] OR doctor*[tiab] OR nurse*[tiab] OR obstetric*[tiab] OR neonatol*[tiab] OR gynecol*[tiab]))

*Limits applied*: English language; publication years 2015–2025

Search strategies were subsequently adapted to the controlled vocabularies and indexing systems of CINAHL, APA PsycINFO, Web of Science, and Psychology and Behavioral Sciences Collection, while maintaining a conceptually consistent structure across databases. All search strategies were developed and verified in collaboration with an expert health sciences librarian to ensure accuracy and comprehensiveness. The final search was conducted in July 2025.

The full search strategies for all databases are provided in Supplementary File S1.

### Selection of sources of evidence

The selection process was conducted in two stages—title and abstract screening followed by full-text assessment—using the CADIMA platform, which also managed the automatic removal of duplicates. Two reviewers (AB, AN) independently screened all records at both stages, following a preliminary calibration exercise designed to ensure consistent application of the eligibility criteria. Any disagreements between the two reviewers were resolved through consultation with a third reviewer (EF).

Eligibility was assessed according to the predefined PCC-based criteria, which focused on the involvement of healthcare professionals in either prenatal counselling or perinatal care, the presence of ethical or moral dimensions related to periviability, and the inclusion of empirical primary research published in English. Studies were excluded when they did not meet one or more of these criteria or when they did not constitute primary research (e.g., theoretical papers, commentaries, editorials). The most frequent reasons for exclusion included lack of an empirical study design and inadequate alignment with the population or conceptual focus of the review.

**Fig. 1:**
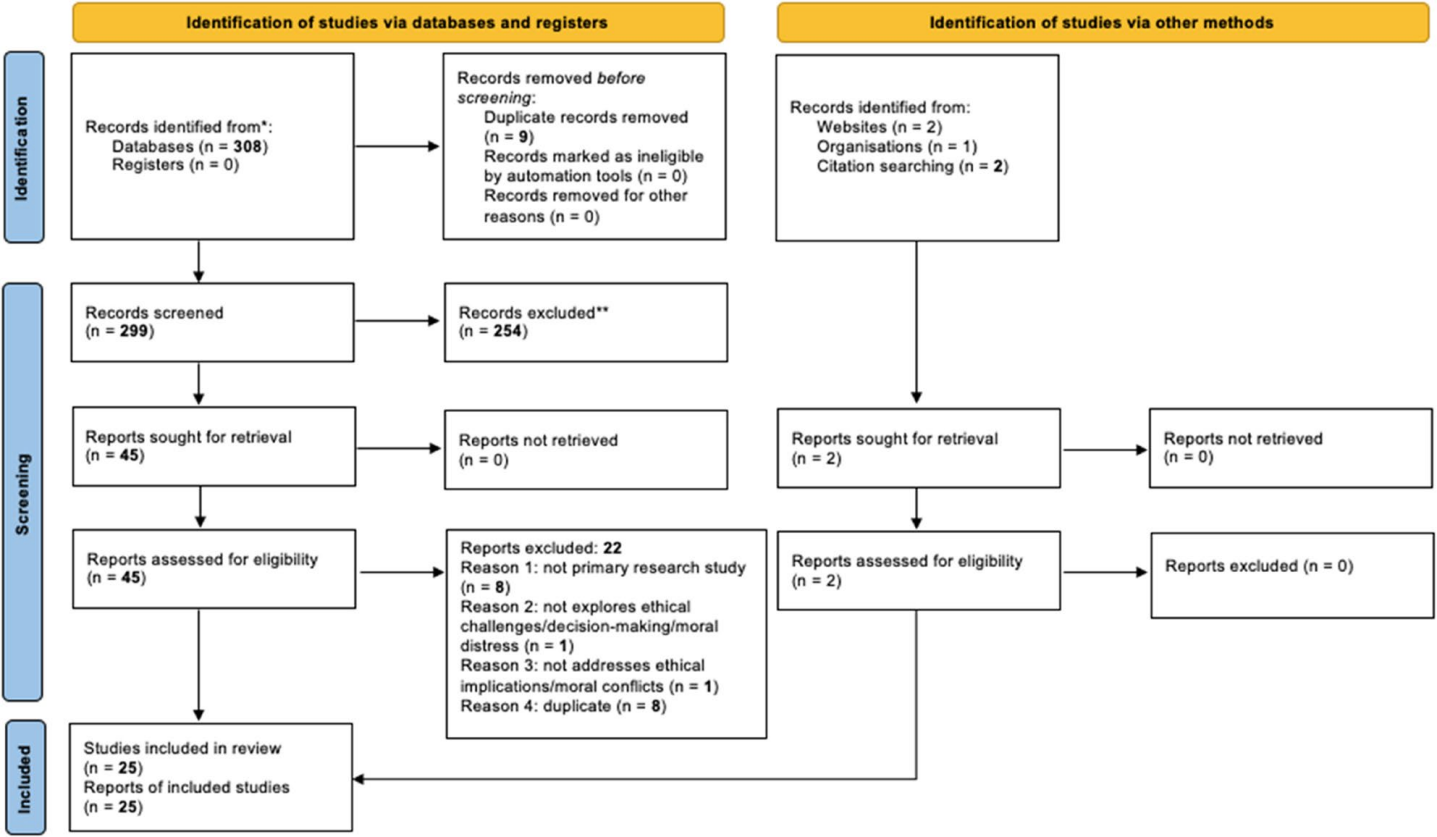
PRISMA 2020 flow diagram for the scoping review

The full selection process, including the number of records identified, screened, included, and excluded at each stage, is illustrated in the PRISMA flow diagram (Fig. 1).

### Data charting process

Data charting was conducted using the CADIMA platform, which enabled standardised extraction and management of information from all included studies. Two reviewers independently charted the data, ensuring accuracy and consistency across the extraction process. Prior to beginning full data charting, the review team conducted a calibration exercise to refine the extraction form and align reviewers’ understanding of each field. This preliminary step involved pilot testing the form on a subset of included studies and discussing discrepancies or uncertainties until consensus was reached, thereby ensuring a coherent and uniform approach to data extraction.

The final data extraction form included the following fields: study ID, country, study design, clinical setting, study objective, healthcare professionals involved, presence or role of CEC, key results or conclusions relevant to ethical issues in periviability counselling, and appraisal information. No authors of primary studies were contacted for clarification or additional information, as the data reported in the included articles were sufficient to meet the aims of the review.

### Data items

For each included study, a predefined set of data items was extracted using a structured form within the CADIMA platform. The variables collected were selected to reflect the objectives of the review and to ensure consistent characterisation of the evidence across heterogeneous study designs.

The following data items were charted: a unique Study ID assigned by the review team (ranging from S01 to S25); the country in which the study was conducted; the study design (qualitative, quantitative, mixed-methods, or randomised controlled trial); and the clinical setting, categorised as MFM, NICU, or other relevant environments. Information on the stated objective of each study was extracted, along with detailed data on the types of healthcare professionals involved and their professional categories. When applicable, the presence, use, or role of CEC was documented. Key results and conclusions relevant to ethical issues, moral conflicts, or decision-making in periviability counselling were also collected. Finally, each study underwent methodological appraisal using the appropriate JBI critical appraisal checklist [45], and the appraisal outcome was recorded.

No assumptions or simplifications were made beyond the information explicitly reported in each study, and only data relating to ethical aspects were extracted. No additional demographic or sample characteristics were charted, as these were outside the scope of the review.

### Critical appraisal of individual sources of evidence

In accordance with the JBI methodology [45], a critical appraisal of all included studies was undertaken using the JBI critical appraisal tools appropriate to each study design. Specifically, the following instruments were applied: the JBI Checklist for Analytical Cross-Sectional Studies, the JBI Checklist for Qualitative Research, the JBI Checklist for RCT, and the JBI Checklist for Mixed Methods Studies. The purpose of this appraisal was not to exclude studies but rather to contextualise the findings by identifying methodological strengths, limitations, and the overall quality of the available evidence. This approach aligns with the JBI guidance for scoping reviews, where critical appraisal is intended to inform interpretation rather than guide study inclusion or exclusion.

### Synthesis of results

The charted data were synthesised using a descriptive and narrative approach, consistent with the exploratory purpose of the review and the heterogeneity of study designs, settings, and outcomes. Quantitative data from cross-sectional surveys and mixed-methods studies were summarised using descriptive statistics, including frequencies and proportions of study characteristics (e.g., country, design, setting, professional groups) and, where available, quantitative indicators related to ethical constructs—such as whether CEC was available, actually used, or reported as needed by professionals—as well as reported moral conflicts or perceived thresholds of viability. These data were tabulated to provide an overview of the distribution of study features across the included evidence base.

Qualitative findings were synthesised through an inductive thematic analysis, which enabled the identification of recurring ethical implications and patterns across diverse professional groups and clinical contexts. The themes derived from this analysis—such as variability in policies and GA thresholds, parental autonomy, prognostic uncertainty, value and religious conflicts, communication challenges, intra-team disagreements, justice and resource allocation, legal responsibility, and moral distress—were developed iteratively by reviewing the extracted data and comparing conceptual patterns across studies.

Findings from qualitative, quantitative, and mixed-methods studies were then integrated narratively to provide a convergent synthesis. This approach allowed the qualitative themes to contextualise and explain heterogeneity observed in the quantitative data, particularly regarding variability in counselling practices, use of CEC, interprofessional differences, and institutional influences. No meta-analysis or formal pooling of data was undertaken due to methodological diversity, and no formal assessment of heterogeneity or publication bias was performed. Variations across study design, geographical context, clinical setting — from MFM services to NICUs — and professional groups were examined descriptively when relevant. The synthesis emphasised mapping the breadth of available evidence, identifying recurrent ethical challenges, and highlighting gaps requiring further empirical investigation.

## Results

### Selection of sources of evidence

A total of 308 records were identified through database searching, with no additional records retrieved from registers. After removal of 9 duplicates, 299 records were screened by title and abstract, of which 254 were excluded for not meeting the eligibility criteria. Forty-five full-text reports were then assessed for eligibility. Of these, 22 were excluded for reasons including lack of primary empirical research (*n* = 8), insufficient focus on ethical dimensions (*n* = 2), or duplicate publications (*n* = 8). Five additional records were identified through other sources (websites *n* = 2, organisations *n* = 1, citation searching *n* = 2). After screening, two reports were retrieved and assessed for eligibility, and both were included in the review. All potentially eligible items were retrieved when available and assessed for eligibility; none were excluded at full-text review.

Ultimately, 25 studies met the eligibility criteria and were included in the review. The full selection process, including identification, screening, eligibility assessment, and reasons for exclusion at each stage, is illustrated in the PRISMA flow diagram (Fig. 1).

### Characteristics of sources of evidence

A total of 25 studies published between 2015 and 2025 were included, encompassing qualitative (*n* = 9), quantitative (*n* = 13), and mixed-methods designs (*n* = 3). The studies were conducted across a wide range of geographical contexts, including North America, Europe, South America, Asia, the Middle East, and sub-Saharan Africa. Most were situated in NICUs or MFM/antenatal settings, while a smaller number were embedded in residency programs, multidisciplinary perinatal environments, or educational and simulation contexts.

Across studies, objectives varied but consistently focused on healthcare professionals’ attitudes, experiences, and ethical dilemmas related to prenatal counselling, thresholds for resuscitation, and decision-making for infants at the threshold of viability. Participant groups were diverse and included neonatologists, obstetricians, MFM specialists, pediatric residents, nurses, midwives, hospitalists, respiratory therapists, and ethics consultants. Ethical issues were examined either directly or indirectly in all studies. Only one study (S01) described the active use of a CEC service, while others discussed ethical concerns conceptually or through participants’ reflections [46].

Common ethical themes emerging across the included studies were prognostic uncertainty, variability in viability thresholds, tensions surrounding parental autonomy, moral distress, value-laden communication, interdisciplinary disagreements, and the influence of institutional culture on counseling practices. Despite the existence of guidelines in some countries, studies consistently documented wide heterogeneity in counseling approaches and clinical decision-making, both within and between professional groups. Physicians tended to emphasise survival data—such as GA-specific survival rates—and broader prognostic assessment incorporating anticipated long-term outcomes, risks of impairment, and clinical responsibility, whereas nurses often foregrounded infant suffering, family experience, and relational ethics. Differences between obstetric and neonatal perspectives, and between senior and junior clinicians, were recurrent, reflecting divergent ethical priorities and varying experiences of moral distress.

Studies conducted in low-resource contexts highlighted additional ethical dilemmas associated with resource scarcity, cultural and religious norms, hierarchical team structures, and constraints on parental involvement [47]. Educational and simulation-based studies underscored the importance of targeted training to enhance communication skills, ethical awareness, and value-sensitive counseling in periviability scenarios [48, 49].

Overall, the included sources of evidence illustrate that ethical challenges in periviability care are pervasive across settings and shaped by clinical uncertainty, interpersonal dynamics, professional norms, and wider systemic factors. These characteristics, and their relevance to the review questions, are summarized in Table 1.

**Table 1:** Characteristics of included sources of evidence

Study ID	Citation	Country	Design	Setting	Objective	Professionals involved	CEC involvement	Key ethical findings
S01	Eves et al. (2015) [46]	USA	Qualitative (ethnography)	NICU	Explore clinicians’ and ethics consultants’ perspectives on neonatal decision-making	Clinicians; ethics consultants	Yes (operational use)	Tensions between prognosis, parental values, and ethical recommendations; CEC supported dialogue and value clarification, though integration was inconsistent.
S02	Ambrósio et al. (2016) [48]	Brazil	Quantitative (survey)	Simulation/training program	Assess instructors’ attitudes toward periviability resuscitation	Neonatal resuscitation instructors	Not reported	Decisions shaped by cultural, legal, and moral norms; marked variability in thresholds and ethical reasoning.
S03	Kukora et al. (2016) [50]	USA	Quantitative (survey)	Residency program; NICU	Examine pediatric residents’ knowledge and attitudes regarding resuscitation thresholds	Pediatric residents	Not reported	Ethical concerns regarding autonomy, equity, pessimism about outcomes, and moral distress; knowledge gaps influenced counseling.
S04	Einaudi et al. (2015) [51]	France	Qualitative (interviews)	NICU	Explore ethical challenges in neonatal end-of-life decision-making	Physicians (obstetrics, neonatology, neurology)	Conceptual	Strong prognostic uncertainty; variability in practices; tension between medical responsibility and parental involvement.
S05	Bucher et al. (2018) [52]	Switzerland	Quantitative (survey)	NICU	Assess perspectives on decision-making at the limit of viability	Neonatologists; NICU nurses	Conceptual	Physicians prioritized prognosis and survival; nurses focused on suffering and family perspective; interdisciplinary ethical tensions evident.
S06	Sperling et al. (2024) [53]	Israel	Quantitative (survey)	NICU	Assess neonatologists’ attitudes toward resuscitation thresholds	Neonatologists	Not reported	Heterogeneity in practice; ethical concerns related to equity, consistency, and guideline adherence.
S07	Dicky et al. (2022) [54]	France	Mixed-methods	NICU	Explore neonatal counseling practices	Neonatologists	Not reported	Ethical conflicts linked to communication challenges, autonomy, and medical authority; discrepancies between parental expectations and clinical communication.
S08	Aujoulat et al. (2018) [55]	Belgium	Quantitative (survey)	NICU	Explore practices regarding periviable care	Neonatologists	Conceptual	Wide variability across NICUs; prognostic uncertainty; inconsistent parental involvement; concerns regarding justice and equity.
S09	Di Stefano et al. (2021) [56]	UK	Quantitative (survey)	NICU	Assess providers’ views on resuscitation and alignment with guidelines	Neonatal professionals	Not reported	Persistent variability despite guidelines; tensions between recommendations and bedside practice; role-based moral distress.
S10	Stanak et al. (2019) [57]	Austria	Qualitative (interviews)	NICU	Explore decision-making at the limit of viability	Neonatologists; ethicist	Ethics expertise present (not formal CEC)	Moral conflicts, uncertainty regarding viability thresholds, and strong influence of institutional norms.
S11	Lawrence (2021) [58]	USA	Quantitative (survey)	MFM/antenatal	Assess providers’ perspectives on counseling and treatment thresholds	Obstetricians; neonatologists; trainees; nurses; respiratory therapists	Conceptual	Divergent perspectives across specialties; obstetricians more autonomy-oriented; ethical dilemmas in counseling evident.
S12	Adams et al. (2025) [59]	USA	Qualitative (interviews)	Mixed perinatal context	Examine institutional culture in periviability care	MFM and neonatal clinicians; nurses; respiratory therapists; hospitalists	Not reported	Moral distress vs. moral resilience; institutional memory outweighing data; interdisciplinary tensions; culture shaping ethics.
S13	Papadimitriou et al. (2019) [49]	Switzerland	RCT (simulation-based training)	Academic simulation center	Evaluate impact of simulation on counseling skills	Medical students; physicians; nurses	Conceptual	Improved communication and ethical awareness; enhanced ability to convey uncertainty; ethics embedded in training.
S14	Schneider et al. (2023) [60]	Germany	Quantitative (survey)	MFM/antenatal	Assess obstetricians’ counseling practices and ethical influences	Obstetricians	Not reported	Counseling shaped by law and institutional policy; variability in communication of outcomes; autonomy–authority dilemmas.
S15	Daboval et al. (2016) [61]	Canada	Qualitative	Antenatal/MFM	Explore factors influencing parental satisfaction in shared decision-making	Neonatologists; fellows; parents	Conceptual	Difficulties in shared decision-making; parental vulnerability; clinician moral distress; communication concerns.
S16	Meadow et al. (2024) [62]	USA	Quantitative (survey)	NICU	Assess staff perspectives on thresholds and parental authority	Physicians; NPs; nurses; hospitalists	Conceptual	Differences across professions; nurses emphasized autonomy; physicians favored intervention; widespread moral conflict.
S17	Rent et al. (2022) [47]	Ethiopia & Ghana	Qualitative (FGDs/interviews)	Neonatal/maternity units	Explore ethical dilemmas in low-resource neonatal care	Physicians; trainees; nurses; midwives	Not reported	Ethical tensions shaped by resource scarcity, cultural norms, hierarchy, and constraints on parental involvement.
S18	Kim et al. (2023) [63]	USA	Quantitative (survey)	MFM + NICU	Compare attitudes between MFM specialists and neonatologists	MFM specialists; neonatologists	Not reported	Divergent counselling priorities; differences in perceived parental authority; variable thresholds for intervention.
S19	Cavolo et al. (2021) [64]	Belgium	Qualitative	NICU	Explore ethical dilemmas in neonatal end-of-life care	Neonatologists	Not reported	Tensions related to prognosis, parental pressure, and moral distress; variability in withdrawal/withholding practices.
S20	Silberberg et al. (2018) [65]	Argentina	Quantitative (survey)	NICU	Assess practices and attitudes toward extremely preterm infants	Neonatologists	Not reported	Wide variability in resuscitation thresholds; limited parental involvement; strong influence of institutional culture.
S21	Tucker Edmonds et al. (2015) [66]	USA	Qualitative	MFM + NICU	Examine approaches to periviability counseling	Obstetricians; neonatologists	Not reported	Ethical tensions in communication; balancing honesty and hope; variable weight assigned to parental preferences.
S22	Arzuaga et al. (2016) [67]	USA	Quantitative (survey)	National	Assess ethical perspectives of Muslim physicians	Physicians	Not reported	Religious values shaped decision-making; reluctance toward withdrawal of care; heterogeneity based on training.
S23	Campo-Engelstein et al. (2024) [68]	USA	Qualitative	MFM/antenatal	Identify ethical dilemmas in periviability counseling	Obstetricians; MFMs; neonatologists	Not reported	Moral distress; conflicts between autonomy and duty; communication dilemmas; institutional policies influenced counseling.
S24	Wang et al. (2023) [69]	China	Quantitative (survey)	National (tertiary hospitals)	Assess attitudes toward counseling and management of periviable births	Obstetricians; neonatologists	Not reported	High variability in thresholds; strong influence of policy and culture; differences across specialties.
S25	Curković et al. (2024) [70]	Croatia	Quantitative (survey)	NICU	Assess ethical reasoning and stress in periviability care	Physicians; nurses	Not reported	Profession-specific ethical tensions; nurses reported higher distress; institutional norms influenced decisions.

### Critical appraisal within sources of evidence

Critical appraisal was conducted for all included studies using the appropriate JBI Critical Appraisal Checklists, selected according to study design (qualitative research, analytical cross-sectional studies, mixed-methods, and the single RCT). Appraisal was performed independently by two reviewers, with disagreements resolved by consensus. In line with the aims of a scoping review, no study was excluded based on methodological limitations; however, appraisal outcomes informed the interpretation and relative weight assigned to the evidence.

Overall, 15 studies were assessed as “Include”, indicating adequate methodological quality, whereas 10 were rated as “Include with reservations”, reflecting moderate limitations that required caution in interpretation. Survey-based cross-sectional studies frequently relied on self-reported attitudes using instruments whose development, piloting, or validation was incompletely described. Confounding factors were seldom fully addressed, and multivariable analyses were uncommon. Several surveys also lacked clarity regarding inclusion criteria, sampling strategies, and representativeness, raising concerns about self-selection and non-response bias.

Across multiple studies, ethically relevant constructs—such as viability thresholds, attitudes toward resuscitation, or perceptions of parental involvement—were measured using non-standardized or unvalidated tools, resulting in partial or uncertain reliability. Mixed-methods and scenario-based studies tended to be stronger, particularly where structured simulation scenarios were used or where appropriate multivariable analyses (e.g., logistic regression) were applied. Qualitative interview studies generally provided rich data and illustrative quotations, but often lacked detailed reflexive accounts or explicit articulation of the researchers’ theoretical lens, which may limit interpretive transparency and transferability.

Recurrent methodological limitations across the evidence base included limited reporting of survey instrument validation, incomplete identification and control of confounders, risks of selection and non-response bias, restricted transferability of single-site qualitative studies, and variable detail regarding ethical approval, especially in older or more conceptually oriented studies.

Despite these limitations, all included studies contributed relevant data for mapping ethical issues, moral dilemmas, and decision-making processes in periviability care. Appraisal findings were integrated narratively into the synthesis to contextualise methodological strengths and limitations without excluding conceptually valuable sources.

### Results of individual sources of evidence

The 25 included studies provided heterogeneous but complementary insights into how healthcare professionals perceive ethical implications, moral conflicts, and decision-making processes in periviability contexts. Charted data from quantitative studies largely described geographical distribution, professional roles, GA thresholds, reported counselling practices, and attitudes toward resuscitation. In contrast, qualitative and mixed-methods sources contributed in-depth accounts of ethical tensions, communication dynamics, and institutional influences shaping practice.

Across individual studies, substantial variation emerged in clinical practices and counselling approaches. Survey data indicated wide discrepancies in GA thresholds for initiating or withholding resuscitation—typically spanning 22–25 weeks—and these differences were shaped by institutional culture, local guidelines, professional background, and national legal frameworks. Such variability often translated into heterogeneous counselling strategies, with some professionals favoring directive guidance and others attempting more collaborative or shared decision-making. Several studies noted inconsistencies between national recommendations—where these existed—and bedside decisions, particularly in contexts where guidelines were absent, ambiguous, or interpreted differently among clinicians.

Qualitative studies consistently highlighted the moral complexity inherent in counselling under conditions of prognostic uncertainty. Professionals described challenges in balancing honesty and hope when discussing survival and long-term outcomes with parents, and in aligning clinical judgment with parental values and expectations. Divergent moral intuitions within clinical teams were also frequently reported: neonatologists tended to place particular emphasis on prognosis and survival probabilities; obstetric and MFM clinicians prioritised maternal risk and antenatal decision-making; nurses foregrounded infant suffering, relational ethics, and the emotional burden experienced by families. These differing perspectives often coexisted within the same institution, contributing to inconsistent messages and occasional intra-team conflict.

Value-laden dilemmas were recurrent across individual studies. Clinicians and families frequently brought cultural norms, religious beliefs, and personal values into the counselling encounter, shaping interpretations of viability and influencing decisions at the threshold of life. These factors intersected with prognostic uncertainty and institutional norms, sometimes generating moral distress—particularly among nurses, junior professionals, and those working in low-resource settings. Although only two studies quantitatively measured moral distress or burnout, many more provided qualitative descriptions of emotional strain and ethical discomfort.

Communication challenges were also evident across individual sources. Interview-based studies and the simulation RCT highlighted difficulties in communicating uncertainty transparently, adapting information to parental needs, and maintaining empathy in emotionally charged conversations. Simulation-based training appeared to enhance communication skills and ethical awareness, yet routine use of structured training or decision-support tools was rarely reported. In several studies, the absence of shared frameworks for counselling contributed to variation in content, tone, and decision-making style.

Institutional culture emerged as a central determinant of decision-making across the included studies. Charted data showed that informal norms, organisational memory, and local professional hierarchies often shaped counselling more strongly than formal guidelines or empirical evidence. How teams interpreted viability thresholds, valued parental involvement, or resolved intra-team disagreements was frequently influenced by local history and context. Studies from low-resource settings underscored additional constraints, including limited technological capacity, hierarchical decision-making structures, and sociocultural expectations surrounding reproduction and parental authority.

With respect to CEC, charted data revealed a notable gap: seventeen studies did not mention CEC, seven referred to it only conceptually or as desirable support, and only one study described operational use in practice. In this example, ethics consultants facilitated dialogue, mediated value conflicts, and supported communication across teams, though integration into routine care remained inconsistent. Taken together, these findings indicate that despite pervasive ethical tensions in periviability care, structured ethics support is rarely embedded in clinical practice.

Overall, the data extracted from individual sources portray a fragmented and context-dependent landscape characterised by variability in thresholds and counselling practices, prognostic uncertainty, value differences, communication challenges, intra-team tensions, and limited access to formal ethics support. These charted findings form the empirical basis for the thematic synthesis presented in the subsequent section.

A quantitative summary of study designs, settings, geographical distribution, professional groups, CEC reporting, and critical appraisal outcomes is provided in Table 2.

**Table 2:** Quantitative characteristics of included studies (*n* = 25)

Domain	Results
Study designs	Cross-sectional surveys 56% (14); qualitative studies 36% (9); mixed-methods 4% (1); randomized controlled trial 4% (1)
Settings	NICU 48% (12); MFM/antenatal 16% (4); mixed perinatal settings 12% (3); other/training/LMIC contexts 24% (6)
Geographical distribution	Europe 40% (10); USA 36% (9); Latin America 8% (2); Asia 8% (2); Africa 4% (1); Canada 4% (1)
Professionals represented	Neonatologists 20; nurses 8; obstetricians/MFM clinicians 7; trainees/fellows 6; ethicists 2; midwives 1
CEC reporting	Not reported 68% (17); conceptual only 28% (7); operational use 4% (1)
JBI appraisal	Include 60% (15); Include with reservations 40% (10)

### Synthesis of results

#### Overview of the Evidence Base

The synthesis of the charted data reveals a complex and heterogeneous landscape in how healthcare professionals perceive ethical challenges and moral conflicts during prenatal counselling at the threshold of viability. Although the 25 included studies differed substantially in design, geography, setting, and professional composition, their findings converged in depicting counselling as an ethically intense, value-laden, and institutionally variable process.

#### Descriptive patterns across study designs and settings

Quantitative mapping showed that most studies were cross-sectional surveys (56%, 14/25), followed by qualitative designs (36%, 9/25), and only two studies adopted mixed-methods or interventional approaches (8%). Nearly half of the studies took place in NICUs (48%, 12/25), with fewer situated in MFM/antenatal contexts (16%, 4/25) or mixed perinatal environments (12%, 3/25). Europe (40%, 10/25) and the USA (36%, 9/25) accounted for many settings, with limited representation from low- and middle-income countries (LMICs) (S17) [47]. Professional samples were dominated by neonatologists (20/25), while nurses (8/25), obstetric/MFM specialists (7/25), trainees (6/25), and ethicists (2/25) were less frequently included.

#### Variability in resuscitation thresholds and counselling practices

A central finding across designs was the marked heterogeneity in resuscitation thresholds, typically ranging between 22 and 25 weeks [53, 55, 56, 65, 69]. These thresholds varied not only internationally but also between neighboring institutions or services within the same hospital. Such variability was mirrored in counselling practices: some clinicians described directive model approaches based on prognosis or institutional norms [52, 63], while others reported attempts to implement shared decision-making [58, 64, 66]. However, even where SDM was invoked conceptually, its consistent application remained limited (only 8/25 studies discussed SDM explicitly).

#### Prognostic uncertainty and ethical tension

Prognostic uncertainty —particularly regarding survival probabilities and long-term neurodevelopmental outcomes—emerged as one of the most pervasive drivers of ethical conflict (10/25). Clinicians frequently commented on the difficulty of communicating ambiguous or incomplete prognostic data [51, 61]. Balancing honesty and hope was perceived as especially challenging when parental expectations diverged from clinical assessments. Qualitative accounts illustrated how uncertainty shaped not only parental decision-making but also clinicians’ sense of moral responsibility, sometimes triggering emotional discomfort or self-doubt.

#### Value pluralism and interprofessional divergence

Conflicts arising from personal, cultural, or religious values were described in 15/25 studies. These value tensions often intersected with prognostic uncertainty, influencing recommendations and interpersonal dynamics within teams [46, 57, 66]. Neonatologists generally prioritised outcome-focused reasoning; obstetric/MFM clinicians highlighted maternal health and autonomy; nurses emphasised the infant’s suffering and relational aspects of care [52, 62, 63]. These divergent orientations contributed to inconsistent messages delivered to parents and occasional intra-team disagreement (8/25).

#### Communication Challenges in High-Stakes Encounters

Communication difficulties constituted a recurrent theme, with 7/25 studies reporting issues related to clarity, tone, or emotional management during counselling [49, 54, 68]. Professionals described struggles in conveying complex prognostic information under time pressure and adapting explanations to parental needs. The only RCT in the dataset showed that simulation-based training improved communication skills and ethical awareness [49], but such structured training was not widely implemented across settings.

#### Institutional culture, policies, and system-level influences

Institutional culture was consistently identified as a determinant shaping counselling and decision-making. Differences in organisational ethos, leadership preferences, historical practices, and available resources influenced both thresholds and communication styles [59, 60, 69]. Studies from LMICs settings [47] highlighted additional constraints, including hierarchical structures, limited parental involvement, and resource-driven ethical dilemmas, underscoring the situated nature of periviability decision-making.

#### Clinical ethics consultation: largely absent despite ethical complexity

The use of CEC was strikingly limited. Seventeen studies made no mention of CEC (68%), seven discussed it only conceptually (28%), and only one study described its operational use in periviability cases [46]. In that context, CEC facilitated dialogue, mediated value conflicts, clarified ethical considerations, and helped address moral distress. The near-complete absence of empirical reporting on CEC represents a significant gap, given the pervasive ethical challenges documented across studies.

#### Moral distress and emotional burden among professionals

Although only two studies quantitatively measured moral distress (8%), qualitative data showed moral distress as a recurrent experience across professions. Nurses and junior clinicians frequently reported emotional strain when clinical duties or institutional norms conflicted with personal moral judgments [59, 64]. Senior professionals described strategies of moral resilience, but institutional mechanisms for supporting staff were largely absent.

#### Integrated interpretation: quantitative heterogeneity explained by qualitative insights

Integrating quantitative and qualitative findings reveals that the observed variability in thresholds, counselling practices, and professional perspectives is not incidental but reflects deeper ethical, cultural, and institutional determinants. Quantitative data documented the extent of heterogeneity—for example in thresholds, SDM practices, and the near-absence of CEC—while qualitative evidence explained the mechanisms underlying this variation, including value pluralism, prognostic uncertainty, institutional culture, and communication constraints. Together, these dimensions form a coherent explanatory framework for understanding the ethical complexity of periviability counselling.

This joint interpretation supports the meta-inference that any meaningful standardisation of periviability counselling must move beyond clinical algorithms alone and incorporate structured ethical reflection, value-sensitive communication strategies, interprofessional alignment, and access to formal ethics support. Such integration appears essential to reduce unwarranted variability and to promote ethically coherent, family-centred decision-making across settings.

#### Gaps, limitations, and future needs

Several gaps were identified. Empirical evaluation of CEC is almost absent, preventing assessment of its effectiveness. Mixed-methods and interventional research remain rare. Midwives, ethicists, psychologists, and parental voices are vastly underrepresented, limiting the scope of perspectives captured. LMIC contexts are insufficiently explored despite distinctive constraints. Finally, outcomes central to ethical practice—consistency of counselling, parental understanding, quality of SDM, moral distress—remain poorly standardised. These gaps point toward the need for more rigorous, interdisciplinary, and context-sensitive research.

A consolidated joint display integrating quantitative patterns, profession-specific emphases, illustrative qualitative insights, and the presence or role of CEC across macro-themes is provided in Table 3. The degree of profession-specific emphasis was derived inductively from the frequency and salience with which particular professional perspectives were reported across the included studies.

**Table 3:** Integrated synthesis of findings across studies: Themes × Professional perspectives × CEC

Theme	Quantitative patterns	Professional perspectives	Illustrative qualitative insight	CEC presence / value	Interpretive synthesis / Identified gap
Principles and values	Value conflicts: 15/25 (60%) Best interests: 4/25 (16%)	Neonatologists ✓✓; OB/MFM ✓; Nurses ✓	“The transcendent meaning of life drives active treatment.” (S20)	Mostly absent; clarifies values in S01	Convergence around value pluralism; gap = operational CEC
Autonomy and decision processes	Parental autonomy: 18/25 (72%) GA thresholds: 17/25 (68%)	OB/MFM ✓✓; Neonatologists ✓; Nurses ✓	“Value elicitation occurred in 1/28 cases.” (S21)	Absent or conceptual	SDM often declared but seldom realised; gap = structured tools
Moral experiences and institutional culture	Moral distress: 2/25; Communication issues: 7/25 (28%)	Nurses ✓✓; OB/MFM ✓; Neonatologists ✓	“Nurses face a higher risk of moral injury.” (S12)	S01: mediation, debriefing, distress mitigation	Moral distress prevalent; gap = structured support/CEC
Cultural, legal, and resource contexts	Justice: 7/25 (28%); Legal aspects: 4/25 (16%)	All professions; strong HIC/LMIC differences	“In Argentina, a liveborn infant is considered a legal subject.” (S20)	Absent or conceptual	Marked heterogeneity in norms and resources; gap = LMIC evidence and CEC evaluation

*GA* Gestational age, *OB* Obstetricians, *SDM* Shared decision-making, *HIC/LMIC *High-/low-income countries

✓✓ = strong emphasis across multiple studies or explicitly highlighted in qualitative findings; ✓ = present but less prominent

## Discussion

This scoping review mapped current evidence on the ethical implications and moral conflicts perceived by healthcare professionals involved in prenatal counselling at the threshold of viability. Across 25 studies conducted in diverse geographical and institutional contexts, a consistent picture emerged of a field marked by substantial variability, entrenched prognostic uncertainty, and profound ethical complexity. The findings directly address the review objectives, showing how ethical tensions are generated not only by clinical parameters but also by institutional cultures, interprofessional dynamics, and value-laden interactions with families. A prominent result of this review is the heterogeneity in resuscitation thresholds and counselling approaches documented across quantitative studies. Decisions regarding initiation or non-initiation of resuscitation between 22 and 25 weeks varied both internationally and within single countries or institutions, reflecting differences in guideline interpretation, medico-legal constraints, resource availability, and professional norms [48, 50, 60, 65]. This variability is ethically significant: without shared frameworks, counselling risks inconsistency, perceived inequity, and reduced clarity for families facing crisis decisions. Several survey studies have examined neonatologists’ preferences regarding resuscitation thresholds or guideline structures, highlighting variability in institutional practices and clinicians’ attitudes toward standardisation [71, 72]. However, these studies generally address clinical decision patterns rather than explicitly analysing the ethical dimensions or moral experiences underpinning such decisions. This distinction underscores the need for research that moves beyond descriptive practice variation to examine the ethical processes shaping counselling and decision-making.

Qualitative and mixed-methods studies helped elucidate the mechanisms underlying this heterogeneity. Prognostic uncertainty emerged as a pervasive influence, making it difficult for clinicians to balance honesty and hope or to communicate complex outcome trajectories in ways meaningful to parents [47, 51, 64]. Uncertainty also intersected with cultural, religious, and personal values. Professionals described how families’ beliefs about suffering, divine will, or the sanctity of life shaped expectations and influenced requests for intervention or comfort-focused care [55, 67, 68]. These encounters also exposed clinicians’ own moral intuitions, sometimes producing value conflicts and emotional burden.

Interprofessional differences further contributed to inconsistent counselling, reflecting the distinct clinical roles and ethical priorities documented across the included studies [52, 58, 63]. These divergent ethical orientations—prognostic, relational, maternal, or suffering-centred—often coexisted within single institutions, generating conflicting messages and occasional intra-team disagreement. When such misalignment occurred, clinicians reported moral distress, frustration, and feelings of compromised integrity [61, 62, 70]. Clinicians frequently described difficulty conveying prognostic uncertainty, eliciting parental values, or tailoring information to families’ emotional readiness. Even when SDM was endorsed, its implementation was often inconsistent, undermined by time constraints, unclear institutional expectations, or conflicting professional assumptions [49, 54]. The limited use of structured communication tools and simulation-based training further contributed to variability. Notably, the absence of clearly defined goals or standardized outcomes for periviability counselling may not solely represent a limitation of current practice but rather reflect the inherently relational and context-sensitive nature of these encounters. In situations characterised by profound uncertainty and value pluralism, counselling goals are often shaped by the specific circumstances, preferences, and moral frameworks of individual families. From this perspective, variability in counselling approaches may not indicate poor practice per se but instead signal attempts to tailor discussions to the needs and values of each family. Accordingly, the quality of counselling may be better conceptualised in terms of procedural features—such as clarity of communication, transparency about uncertainty, alignment with parental values, and opportunities for deliberation—rather than by fixed clinical or decisional outcomes. Recognising this distinction may help explain part of the heterogeneity observed across studies and supports the view that ethically robust counselling in periviability should prioritise process-oriented measures of quality alongside clinical considerations.

A similar emphasis on process rather than predetermined outcomes is also reflected in contemporary frameworks of clinical ethics consultation. Current guidance emphasises that ethics consultation does not rely on a single universally agreed procedural model but is instead understood as a structured facilitation process aimed at guiding discussion among ethically justifiable options and supporting shared deliberation among participants [73]. This parallel further supports the view that ethical quality in complex clinical encounters may be better assessed through the integrity of the deliberative process rather than through uniform decisional outcomes.

Institutional culture emerged as a key determinant of ethical practice. In many settings, local norms, historical patterns, and implicit hierarchies shaped decision-making more strongly than guidelines or empirical evidence. “Institutional memory,” unwritten rules, and unit-specific traditions influenced how viability thresholds were interpreted and how counselling was delivered [57, 59]. In low-resource contexts, ethical challenges were compounded by limited technological capacity, hierarchical structures, and broader sociocultural expectations [47]. Amidst this complex landscape, the near absence of CEC was striking. Only one study described CEC being actively utilised [46], in which ethics consultants supported communication, mediated conflicting values, clarified goals of care, and addressed moral distress. Several studies mentioned CEC conceptually or as a desirable but missing resource, yet none evaluated its processes or outcomes. National surveys in neonatal intensive care settings similarly report variability in access to and utilisation of ethics support services, highlighting persistent gaps in structured ethics integration [74]. This gap is notable given that periviability represents one of the most ethically challenging areas of neonatal and perinatal care. The limited availability and integration of formal ethics support may contribute to unresolved tensions, fragmented counselling, and moral distress across professional groups. Importantly, the limited operational presence of clinical ethics consultation (CEC) in periviability should not be interpreted solely as evidence of impracticality. Contemporary frameworks of healthcare ethics consultation describe CEC as a structured facilitation process aimed at guiding discussion among ethically justifiable options, mediating entrenched conflict, and strengthening the quality of decision-making without displacing clinical authority [73, 75, 76]. In this sense, CEC does not replace clinical judgment but enhances the deliberative process through which morally complex decisions are examined Within this perspective, the ethics consultant occupies a position that is external to the immediate clinical decision but not detached from the deliberative process. Rather than remaining neutral in the sense of disengaged, the consultant participates in facilitating dialogue, helping participants in the deliberation clarify values, articulate reasons, and explore ethically justifiable options. In this sense, the ethics consultant can be understood as external but not extraneous to the clinical team: while maintaining a critical distance from the decision itself, the consultant actively contributes to the deliberative process by fostering trust, clarifying normative tensions, and supporting constructive engagement among stakeholders [75, 76].

In situations of entrenched disagreement, this role may help create the conditions for trust and structured deliberation without displacing clinical authority or parental involvement. In many clinical pathways, opportunities for anticipatory ethics support exist before delivery occurs. Ethics consultation may therefore operate not only as a reactive response to acute disagreement but also as a proactive component of perinatal care pathways, identifying ethically complex situations early in the clinical trajectory [39]. Situations such as threatened preterm labour, preterm premature rupture of membranes, or prolonged antenatal hospitalisation often create a temporal window in which ethical reflection, value clarification, and shared deliberation can be supported prior to urgent decision-making at birth. In this way, CEC may function not only as a bedside intervention but also as a proactive component of prenatal care pathways, helping teams and families prepare for ethically complex scenarios. At the same time, CEC retains its core function as case-based bedside consultation when disagreement, uncertainty, or value conflict arise. Experiences from neonatal intensive care units where structured ethics consultation has been implemented illustrate how case-based consultation can support interdisciplinary deliberation and conflict mediation in complex neonatal cases [77].

However, its role in periviability need not be limited to reactive intervention. Evidence from intensive care settings suggests that proactive ethics consultation and structured ethics processes can be meaningfully embedded within clinical practice, supporting communication, mediating value conflicts, and fostering coherent team deliberation. Such models illustrate that ethics consultation can operate both at the bedside and as an integrated component of organisational practice [39]. Further empirical reports from neonatal intensive care settings suggests that CEC can be meaningfully integrated into clinical practice. Beyond the studies included in this review, empirical reports from NICUs indicate that ethics consultation may facilitate dialogue among professionals, support communication with families, and help navigate conflicts regarding goals of care and treatment limitations. Recent evaluations of NICU-based ethics consultation services further demonstrate feasibility, clinician engagement, and perceived usefulness of structured ethics support in neonatal settings [78]. These models illustrate that ethics support can be embedded within organisational structures rather than activated only in emergencies. Framing CEC as anticipatory, consultative, and system-integrated—rather than solely reactive—may therefore help reconcile its potential value with the practical constraints of periviability care. At the same time, CEC is unlikely to be necessary for all periviability cases and may be most appropriately engaged not simply in the presence of prognostic uncertainty—which is inherent to periviability—but when such uncertainty translates into difficulties in the decision-making process itself. In particular, ethics consultation may be helpful when parents struggle to articulate their values or preferences, when decisional paralysis or marked ambivalence emerges, or when the decision-making process appears constrained by emotional burden or relational tension. Within this perspective, parental values should not be understood as pre-existing and readily accessible, but as emerging through engagement with uncertainty and within relational processes of deliberation. In this sense, decisions are not made despite uncertainty, but through engaging with it. In these situations, the role of CEC is not to determine what decision ought to be made, but to support the conditions under which a meaningful and authentic decision can be reached. Ethics consultation may therefore facilitate the articulation of values, support reflective engagement with uncertainty, and help ensure that decisions are not only made, but meaningfully owned by those involved. In this sense, the aim is not simply to enable individuals to do what they want, but to support them in coming to want what they do. This approach reframes the contribution of CEC from a primarily conflict-resolution function to a process-oriented role that enables deliberation, particularly when uncertainty or emotional burden impairs participants’ ability to engage fully in shared decision-making.

Importantly, consultation is not requested only in the presence of technical doubt, but also when entrenched moral conflict or sustained value divergence require structured facilitation. While gestational cut-offs may provide organisational clarity and medico-legal structure, they cannot in themselves resolve underlying moral disagreement. This distinction has been widely recognised in neonatal ethics literature, where decision-making in the NICU is described as inherently value-laden and not reducible to clinical prognostic data alone [79].

Decisions at the threshold of viability engage normative judgments concerning proportionality, suffering, parental authority and best interest. Where disagreement persists, structured ethical deliberation remains necessary. In this sense, awareness of the availability of ethics consultation may support neonatologists in recognising that complex periviability decisions are not reducible to clinical parameters alone [39].

Taken together, these findings —including the limited yet potentially actionable role of ethics consultation—demonstrate that ethical challenges in periviability are not isolated dilemmas but arise from the intersection of clinical uncertainty, value pluralism, interprofessional divergence, and institutional culture. The review highlights key implications for neonatologists, maternal–fetal medicine clinicians, nurses, midwives, ethics consultants, and healthcare systems more broadly. The evidence suggests that ethically grounded, value-sensitive counselling requires more than technical expertise: it depends on structured communication strategies, coordinated interprofessional practice, routine elicitation of parental values, and the integration of ethics support into clinical pathways. Without such components, counselling remains vulnerable to variability, misalignment, and moral distress.

### Implications for practice

Findings from this review underscore the need to strengthen ethical, communicative, and interprofessional foundations in periviability counselling. Minimum counselling standards—including clear communication of prognosis, explicit discussion of uncertainty, elicitation of parental values, and consistent framing of options—should be standardised while allowing space for contextualised decision-making. SDM can be reinforced through validated tools and structured approaches that help align parental preferences with clinical judgment. Integration of CEC into periviability pathways may be most feasible when conceived as anticipatory, consultative, and system-embedded rather than solely reactive bedside intervention. Ethics support may therefore contribute to early identification of value conflicts, mediation of interprofessional disagreement, facilitation of complex SDM conversations, and mitigation of moral distress. When available, ethics consultation may assist clinicians in urgent situations, supporting deliberation under time pressure without displacing clinical responsibility for decision-making. Activation criteria—such as difficulty in articulating parental values, decisional paralysis or marked ambivalence, or situations where emotional burden or relational tensions constrain meaningful participation in decision-making—could facilitate timely and proportionate engagement of ethics consultation. Such criteria also help ensure that ethics consultation is used proportionately, supporting cases where ethical complexity exceeds routine clinical deliberation rather than functioning as a universal requirement. In situations where conflict arises between clinicians and parents, ethics consultation may play a particularly important role by facilitating dialogue and helping restore conditions for trust and constructive deliberation. In this sense, the ethics consultant remains external to the decision itself while actively supporting the deliberative process, without aligning with any single party but helping participants clarify values, articulate reasons, and work toward shared decisions.

In many cases, ethics input may be particularly valuable during the prenatal phase, when risk of preterm delivery is recognised and time exists for anticipatory deliberation. Investment in training that combines communication skills, ethics reflection, and interprofessional simulation is also needed. Finally, broadening the professional groups involved in counselling to include nursing, midwifery, psychology, and ethics expertise may enhance relational, cultural, and value-sensitive aspects of care.

#### Implications for research

Future research should prioritise rigorous evaluation of CEC models in periviability, examining activation processes, stakeholder experiences, and outcomes for families and staff. Development of core outcome sets for counselling—such as decisional conflict, parental satisfaction, quality of SDM, value-concordance, and moral distress—would strengthen comparability across studies. Prospective and mixed-methods designs capable of linking institutional ethical climate with counselling quality are needed. Addressing geographical and contextual gaps is also essential, particularly in low- and middle-income countries where ethical challenges are shaped by resource constraints and sociocultural dynamics. Research should systematically include under-represented professions—nurses, midwives, ethicists, and psychologists—to better capture the diversity of ethical expertise and lived experience involved in periviability care. Advancing this research agenda will also require greater recognition of neonatal ethics as a priority area for funding and interdisciplinary collaboration. Empirical work in this field often involves small, specialised populations and ethically sensitive contexts, which demand substantial methodological and organisational support. Investment in multicentre and multidisciplinary research collaborations, validated measurement tools, and dedicated funding streams for bioethics research would substantially strengthen the robustness and generalisability of future evidence.

#### Limitations

This scoping review has limitations inherent to its methodology. The evidence base was heterogeneous and dominated by self-reported survey data, often using non-validated instruments and voluntary samples. Qualitative studies were generally robust but frequently lacked reflexivity, constraining interpretive depth. Mixed-methods and interventional studies were rare, limiting triangulation. Representation of key groups—especially midwives, psychologists, and ethicists—was limited, and LMIC contexts remain under-explored. As scoping reviews do not synthesise effect sizes or judge overall study quality, interpretive emphasis lies on conceptual breadth rather than evaluative precision. These limitations should not be interpreted solely as weaknesses of individual studies but also reflect structural challenges in conducting empirical research in neonatal ethics, including limited funding opportunities, small specialised populations, and the sensitivity of studying ethically complex clinical encounters. Strengthening the evidence base will therefore require sustained institutional support, interdisciplinary collaboration, and investment in methodologically rigorous empirical bioethics research. Nonetheless, the convergence of findings across disparate study designs and contexts enhances confidence in the conclusions drawn.

## Conclusions

Periviability counselling remains ethically complex and unevenly implemented across settings. Despite widespread endorsement of parental involvement, genuine SDM is inconsistently realised, challenged by substantial prognostic uncertainty, divergent professional perspectives, and the strong influence of institutional culture. These factors contribute to moral conflict and, for many clinicians—particularly nurses and junior staff—significant moral distress. Clinical ethics consultation (CEC) emerges as a potentially valuable but still underutilised resource, with limited empirical evaluation of its processes, activation criteria, and practical integration into periviability care pathways.

Improving the ethical quality of periviability care will require changes that are both organisational and cultural. On the organisational side, structured ethics support and clear deliberative frameworks may help ensure that complex decisions are not left solely to individual clinicians but are guided by shared processes and accessible expertise when ethical complexity exceeds routine clinical deliberation. At the same time, strengthening communication and shared decision-making skills remains essential, as these competencies enable clinicians to navigate uncertainty, convey complex prognostic information, and build constructive partnerships with families.

Cultural change is equally important. Periviability counselling should not remain the exclusive domain of neonatology but should involve a broader multidisciplinary team including obstetrics and maternal–fetal medicine, midwifery, nursing, psychology, and clinical ethics. Such diversity of perspectives may help reduce professional silos, support coherent communication with families, and foster more relational and value-sensitive counselling practices.

Finally, addressing existing gaps in training, institutional policies, and empirical research is essential for fostering transparent and ethically robust decision-making at the threshold of viability. Advancing this field will also require stronger institutional recognition of neonatal ethics as a research priority, including investment in multicentre and multidisciplinary collaborations, validated measurement tools, and dedicated funding streams capable of supporting rigorous empirical bioethics research. Without sustained efforts in these areas, improvements in ethical practice are likely to remain fragmented and difficult to sustain.

## Supplementary Information

Supplementary Material 1: Supplementary File S1. Full search strategies for all databases.

## Data Availability

All data generated or analysed during this study are included in this published article and its supplementary information files. The data extraction matrix and coding framework used for the synthesis are available from the corresponding author upon reasonable request.

## References

[1] American College of Obstetricians and Gynecologists; Society for Maternal-Fetal Medicine. Obstetric Care consensus 6: Periviable Birth. Obstet Gynecol. 2017;130(4):e187e199. 10.1097/AOG.0000000000002352. 28937572 10.1097/AOG.0000000000002352

[2] Morgan AS, Zeitlin J, Källén K, Draper ES, Maršál K, Norman M, et al. Birth outcomes between 22 and 26 weeks’ gestation in national population-based cohorts from Sweden, England and France. Acta Paediatr. 2022;111(1):59–75. 10.1111/apa.16084. 34469604 10.1111/apa.16084 PMC9291863

[3] Getaneh T, Homaira N, Kasaye H, Tapawan SJC, Chughtai AA, Lui K. Global inequities in the survival of extremely preterm infants: a systematic review and meta-analysis. BMC Pediatr. 2025;25(1):579. 10.1186/s12887-025-05933-w. 40739629 10.1186/s12887-025-05933-w PMC12309206

[4] EPICE, Zeitlin J, Maier RF, Cuttini M, Aden U, Boerch K, Gadzinowski J, et al. Cohort profile: Effective Perinatal Intensive Care in Europe (EPICE) very preterm birth cohort. Int J Epidemiol. 2020;49(2):372–86. 10.1093/ije/dyz270. 32031620 10.1093/ije/dyz270 PMC7266542

[5] Venkatesh KK, Lynch CD, Costantine MM, Backes CH, Slaughter JL, Frey HA, et al. Trends in active treatment of live-born neonates between 22 weeks 0 days and 25 weeks 6 days by gestational age and maternal race and ethnicity in the US, 2014–2020. JAMA. 2022;328(7):652–60. 10.1001/jama.2022.12841. 35972487 10.1001/jama.2022.12841 PMC9382444

[6] Al Gharaibeh FN, Cortezzo DE, Nathan AT, Greenberg JM. The impact of standardization of care for neonates born at 22–23 weeks gestation. J Perinatol. 2025. 10.1038/s41372-025-02214-3. 10.1038/s41372-025-02214-3 39905244

[7] World Health Organization. Born too soon: decade of action on preterm birth. Geneva: World Health Organization; 2023.

[8] Vidaeff AC, Kaempf JW. The ethics and practice of periviability care. Child (Basel). 2024;11(4):386. 10.3390/children11040386. 10.3390/children11040386 PMC11049503 38671603

[9] Hendriks MJ, Lantos JD. Fragile lives with fragile rights: justice for babies born at the limit of viability. Bioethics. 2018;32(3):205–14. 10.1111/bioe.12428. 29369374 10.1111/bioe.12428

[10] Arimitsu T, Hatayama K, Gaughwin K, Kusuda S. Ethical considerations regarding the treatment of extremely preterm infants at the limit of viability: a comprehensive review. Eur J Pediatr. 2025;184(2):140. 10.1007/s00431-025-05976-2. 39814940 10.1007/s00431-025-05976-2

[11] Vidaeff AC, Capito L, Gupte S, Antsaklis A, FIGO Committee on the Ethical Aspects of Human Reproduction and Women’s Health. The ethics and practice of perinatal care at the limit of viability: FIGO recommendations. Int J Gynaecol Obstet. 2024;166(2):644–7. 10.1002/ijgo.15744. 38944691 10.1002/ijgo.15744

[12] Comitato Nazionale per la Bioetica. Atteggiamenti e decisioni alle soglie della nascita. Roma: Presidenza del Consiglio dei Ministri. 2008. Available from: https://bioetica.governo.it/media/3122/p80_2008_grandi_prematuri_it.pdf.

[13] Carta di Roma. La prematurità estrema: margini di gestione ostetrica e risvolti neonatologici. Roma. 2008. Available from: https://www.aslal.it/allegati/Formazione/Corso_bioetica/carta_di_roma.pdf.

[14] Guillén Ú, Suh S, Munson D, Posencheg M, Truitt E, Zupancic JAF, et al. Development and pretesting of a decision-aid to use when counseling parents facing imminent extreme premature delivery. J Pediatr. 2012;160(3):382–7. 10.1016/j.jpeds.2011.08.070. 22048056 10.1016/j.jpeds.2011.08.070

[15] Haward MF, Gaucher N, Payot A, Robson K, Janvier A. Personalized decision making. Clin Perinatol. 2017;44(2):429–45. 10.1016/j.clp.2017.01.006. 28477670 10.1016/j.clp.2017.01.006

[16] Fawke J, Tinnion RJ, Monnelly V, Ainsworth SB, Cusack J, Wyllie J. How does the BAPM Framework for Practice on Perinatal Management of Extreme Preterm Birth Before 27 Weeks of Gestation impact delivery of Newborn Life Support? A Resuscitation Council UK response. Arch Dis Child Fetal Neonatal Ed. 2020;105(6):672–4. 10.1136/archdischild-2020-318927. 32273302 10.1136/archdischild-2020-318927

[17] De Proost L, Geurtzen R, Ismaili M’hamdi, Reiss H, Steegers IKM, Verweij EAP. Prenatal counseling for extreme prematurity at the limit of viability: a scoping review. Patient Educ Couns. 2022;105(7):1743–60. 10.1016/j.pec.2021.10.033. 34872804 10.1016/j.pec.2021.10.033

[18] Janvier A, Lorenz JM, Lantos JD. Antenatal counselling for parents facing an extremely preterm birth: limitations of the medical evidence. Acta Paediatr. 2012;101(8):800–4. 10.1111/j.1651-2227.2012.02695.x. 22497312 10.1111/j.1651-2227.2012.02695.x

[19] Simpkin AL, Armstrong KA. Communicating uncertainty: a narrative review and framework for future research. J Gen Intern Med. 2019;34(11):2586–91. 10.1007/s11606-019-04860-8. 31197729 10.1007/s11606-019-04860-8 PMC6848305

[20] Lemmon ME, Barks MC, Bansal S, Bernstein S, Kaye EC, Glass HC, et al. The ALIGN framework: a parent-informed approach to prognostic communication for infants with neurologic conditions. Neurology. 2023;100(8):e. 10.1212/WNL.0000000000201600. 10.1212/WNL.0000000000201600 PMC9984217 36456199

[21] Cummings J, Committee on Fetus and Newborn, Watterberg K, Eichenwald E, Poindexter B, et al. Antenatal counseling regarding resuscitation and intensive care before 25 weeks of gestation. Pediatrics. 2015;136(3):588–95. 10.1542/peds.2015-2336. 26324869 10.1542/peds.2015-2336

[22] Geurtzen R, van Heijst A, Draaisma J, Ouwerkerk L, Scheepers H, Hogeveen M, et al. Prenatal counseling in extreme prematurity: insight into preferences from experienced parents. Patient Educ Couns. 2019;102(8):1541–9. 10.1016/j.pec.2019.03.016. 30948203 10.1016/j.pec.2019.03.016

[23] Cypher RL, Foglia LM. Periviability: a review of key concepts and management for perinatal nursing. J Perinat Neonatal Nurs. 2020;34(2):146–54. 10.1097/JPN.0000000000000473. 32332444 10.1097/JPN.0000000000000473

[24] Sullivan A, Arzuaga B, Luff D, Young V, Schnur M, Williams D, et al. A qualitative study of parental perspectives on prenatal counseling at extreme prematurity. J Pediatr. 2022;251:17–e232. 10.1016/j.jpeds.2022.09.003. 36096177 10.1016/j.jpeds.2022.09.003 PMC9729443

[25] Peterson J, Graham C, Johnstone ED, Mahaveer A, Smith DM. A rapid review of periviable (22 + 0 to 23 + 6 weeks) counselling practices and the need for a trauma-informed care approach. Front Pediatr. 2025;13:1553040. 10.3389/fped.2025.1640856. 40475219 10.3389/fped.2025.1553040 PMC12137106

[26] Prentice TM, Janvier A, Gillam L, Donath S, Davis PG. Moral distress in neonatology. Pediatrics. 2021;148(2):e2020031864. 10.1136/archdischild-2015-309410. 34285081 10.1542/peds.2020-031864

[27] Lamiani G, Borghi L, Argentero P. When healthcare professionals cannot do the right thing: a systematic review of moral distress and its correlates. J Health Psychol. 2017;22(1):51–67. 10.1177/1359105315595120. 26220460 10.1177/1359105315595120

[28] Trotochaud K, Coleman JR, Krawiecki N, McCracken C. Moral distress in pediatric healthcare providers. J Pediatr Nurs. 2015;30(6):908–14. 10.1016/j.pedn.2015.03.001. 25869472 10.1016/j.pedn.2015.03.001

[29] Kornhauser Cerar L, Lucovnik M. Ethical dilemmas in neonatal care at the limit of viability. Child (Basel). 2023;10(5):784. 10.3390/children10050784. 10.3390/children10050784 PMC10217697 37238331

[30] Mills M, Cortezzo DE. Moral Distress in the Neonatal Intensive Care Unit: What Is It, Why It Happens, and How We Can Address It. Front Pediatr. 2020;8:581. 10.3389/fped.2020.00581. 33014949 10.3389/fped.2020.00581 PMC7511509

[31] Deligianni M, Voultsos P, Tzitiridou-Chatzopoulou MK, Drosou-Agakidou V, Tarlatzis V. Moral distress among neonatologists working in neonatal intensive care units in Greece: a qualitative study. BMC Pediatr. 2023;23(1):114. 10.1186/s12887-023-03918-1. Erratum in: BMC Pediatr. 2024;24(1):312. https://doi.org/10.1186/s12887-024-04802-2. 10.1186/s12887-023-03918-1 PMC9993694 36890500

[32] Prentice T, Janvier A, Gillam L, Davis PG. Moral distress within neonatal and paediatric intensive care units: a systematic review. Arch Dis Child. 2016;101(8):701–8. 10.1542/peds.2020-031864. 26801075 10.1136/archdischild-2015-309410

[33] Ernst P, Linden K, Roczniewska M, Hadzibajramovic E, Wessberg A, Andersson O, et al. Moral distress among maternity and neonatal healthcare workers during the COVID-19 pandemic in Sweden: results from the COPE staff longitudinal cohort study. Acta Obstet Gynecol Scand. 2025;104(7):1399–409. 10.1111/aogs.15125. 40247755 10.1111/aogs.15125 PMC12144571

[34] Epstein EG, Hamric AB. Moral distress, moral residue, and the crescendo effect. J Clin Ethics. 2009;20(4):330–42. PMID: 20120853. 20120853

[35] Rezaei Z, Nematollahi M, Asadi N. The relationship between moral distress, ethical climate, and attitudes towards care of a dying neonate among NICU nurses. BMC Nurs. 2023;22(1):303. 10.1186/s12912-023-01459-7. 37670308 10.1186/s12912-023-01459-7 PMC10478422

[36] Orzalesi MM, Cuttini M. Ethical issues in neonatal intensive care. Ann Ist Super Sanita. 2011;47(3):273–7. 10.4415/ANN_11_03_06. 21952152 10.4415/ANN_11_03_06

[37] DuVal G, Clarridge B, Gensler G, Danis M. A national survey of U.S. internists’ experiences with ethical dilemmas and ethics consultation. J Gen Intern Med. 2004;19(3):251–8. 10.1111/j.1525-1497.2004.21238.x. 15009780 10.1111/j.1525-1497.2004.21238.x PMC1492156

[38] Bell JAH, Salis M, Tong E, Nekolaichuk E, Barned C, Bianchi A, et al. Clinical ethics consultations: a scoping review of reported outcomes. BMC Med Ethics. 2022;23(1):99. 10.1186/s12910-022-00832-6. 36167536 10.1186/s12910-022-00832-6 PMC9513991

[39] Picozzi M, Gasparetto A. Clinical ethics consultation in the intensive care unit. Minerva Anestesiol. 2020;86(6):670–7. 10.23736/S0375-9393.20.14028-8. 32000471 10.23736/S0375-9393.20.14028-8

[40] Stanak M. Neonatology in Austria: ethics to improve practice. Med Health Care Philos. 2020;23(3):361–9. 10.1007/s11019-020-09943-6. 32144643 10.1007/s11019-020-09943-6 PMC7426316

[41] Chen HA, Drago MJ. Professional guidelines for the care of extremely premature neonates: clinical reasoning versus ethical theory. J Clin Ethics. 2023;34(3):233–44. 10.1086/726813. 37831654 10.1086/726813

[42] Göbert P, von Blanckenburg P, Maier RF, Seifart C. Utilization and evaluation of ethics consultation services in neonatal intensive care. Child (Basel). 2024;11(11):1349. 10.3390/children11111349. 10.3390/children11111349 PMC11593247 39594924

[43] Peters MD, Godfrey C, McInerney P, Munn Z, Tricco AC, Khalil H. Scoping reviews. In: JBI manual for evidence synthesis. JBI; 2024. Available from: https://jbi-global-wiki.refined.site/space/MANUAL/355862497/10.+Scoping+reviews. 10.11124/JBIES-20-00167 33038124

[44] Tricco AC, Lillie E, Zarin W, O’Brien KK, Colquhoun H, Levac D, et al. PRISMA extension for scoping reviews (PRISMA-ScR): checklist and explanation. Ann Intern Med. 2018;169(7):467–73. 10.7326/M18-0850. 30178033 10.7326/M18-0850

[45] JBI. JBI Critical Appraisal Tools. Adelaide: JBI. 2020. Available from: https://jbi.global/critical-appraisal-tools.

[46] Eves MM, Danziger PD, Farrell RM, Cole CM. Conflicting values: a case study in patient choice and caregiver perspectives. Narrat Inq Bioeth. 2015;5(2):167–78. 10.1353/nib.2015.0054. 26300149 10.1353/nib.2015.0054

[47] Rent S, Bakari A, Aynalem Haimanot S, Deribessa SJ, Plange-Rhule G, Bockarie Y, et al. Perspectives on resuscitation decisions at the margin of viability among specialist newborn care providers in Ghana and Ethiopia: a qualitative analysis. BMC Pediatr. 2022;22(1):97. 10.1186/s12887-022-03146-z. 35177012 10.1186/s12887-022-03146-z PMC8851801

[48] Ambrósio CR, de Almeida MFB, Guinsburg R. Opinions of Brazilian resuscitation instructors regarding resuscitation in the delivery room of extremely preterm newborns. J Pediatr (Rio J). 2016;92(6):609–15. 10.1016/j.jped.2016.02.012. 27260873 10.1016/j.jped.2016.02.012

[49] Papadimitriou V, Tosello B, Pfister R. Effect of written outcome information on attitude of perinatal healthcare professionals at the limit of viability: a randomized study. BMC Med Ethics. 2019;20(1):74. 10.1186/s12910-019-0413-7. 31640670 10.1186/s12910-019-0413-7 PMC6806555

[50] Kukora S, Laventhal N. Resident attitudes on ethical and medical decision-making for neonates at the limit of viability. Am J Perinatol. 2016;33(5):449–55. 10.1055/s-0035-1565916. 26485248 10.1055/s-0035-1565916

[51] Einaudi MA, Gire C, Auquier P, Le Coz P. How do physicians perceive quality of life? Ethical questioning in neonatology. BMC Med Ethics. 2015;16(1):50. 10.1186/s12910-015-0045-5. 26204881 10.1186/s12910-015-0045-5 PMC4512037

[52] Bucher HU, Klein SD, Hendriks MJ, Baumann-Hölzle R, Berger TM, et al. Decision-making at the limit of viability: differing perceptions and opinions between neonatal physicians and nurses. BMC Pediatr. 2018;18(1):81. 10.1186/s12887-018-1040-z. 29471821 10.1186/s12887-018-1040-z PMC5822553

[53] Sperling D, Riskin A, Borenstein-Levin L, Hochwald O. At the threshold of viability: to resuscitate or not to resuscitate—perspectives of Israeli neonatologists. BMJ Paediatr Open. 2024;8(1):e002633. 10.1136/bmjpo-2024-002633. 10.1136/bmjpo-2024-002633 PMC11097872 38754896

[54] Dicky O, Dahan S, Reynaud A, Goffinet F, Lecarpentier E, Deruelle P, et al. Current attitudes and beliefs toward perinatal care orientation before 25 weeks of gestation: the French perspective. Semin Perinatol. 2022;46(2):151533. 10.1016/j.semperi.2021.151533. 34865886 10.1016/j.semperi.2021.151533

[55] Aujoulat I, Henrard S, Charon A, Johansson AB, Langhendries JP, et al. End-of-life decisions and practices for very preterm infants in the Wallonia-Brussels Federation of Belgium. BMC Pediatr. 2018;18(1):206. 10.1186/s12887-018-1168-x. 29945564 10.1186/s12887-018-1168-x PMC6020374

[56] Di Stefano LM, Wood K, Mactier H, Bates SE, Wilkinson D. Viability and thresholds for treatment of extremely preterm infants: survey of UK neonatal professionals. Arch Dis Child Fetal Neonatal Ed. 2021;106(6):596–602. 10.1136/archdischild-2020-321273. 33927001 10.1136/archdischild-2020-321273 PMC8543207

[57] Stanak M, Hawlik K. Decision-making at the limit of viability: the Austrian neonatal choice context. BMC Pediatr. 2019;19(1):204. 10.1186/s12887-019-1569-5. 31221128 10.1186/s12887-019-1569-5 PMC6585118

[58] Lawrence C, Laventhal N, Fritz KA, Carlos C, Famuyide M, Tonismae T, et al. Ethical cultures in perinatal care: correlation of provider attitudes with periviability practices at six centers. Am J Perinatol. 2021;38(S1):e193–200. 10.1055/s-0040-1709128. 32294770 10.1055/s-0040-1709128

[59] Adams SY, Fry JT, Henner N. What is culture made of? An exploratory study of ethical cultures and provider perspectives on the care of periviable neonates. Am J Perinatol. 2025;42(4):502–10. 10.1055/a-2405-3336. 39299244 10.1055/a-2405-3336

[60] Schneider K, Müller J, Schleußner E. German obstetricians’ self-reported attitudes and handling in threatening preterm birth at the limits of viability. J Perinat Med. 2023;51(8):1097–103. 10.1515/jpm-2022-0547. 37256371 10.1515/jpm-2022-0547

[61] Daboval T, Shidler S, Thomas D. Shared decision making at the limit of viability: a blueprint for physician action. PLoS ONE. 2016;11(11):e0166151. 10.1371/journal.pone.0166151. 27893823 10.1371/journal.pone.0166151 PMC5125593

[62] Meadow J, Arzu J, Rychlik K, Henner N. Trial of therapy on trial: inconsistent thresholds for discussing withdrawal of life-sustaining therapies in the neonatal intensive care unit. Am J Perinatol. 2024;41(S1):e794–802. 10.1055/a-1941-4285. 36096150 10.1055/a-1941-4285

[63] Kim BH, Feltman DM, Schneider S, Herron C, Montes A, Anani UE, et al. What information do clinicians deem important for counseling parents facing extremely early deliveries? Results from an online survey. Am J Perinatol. 2023;40(6):657–65. 10.1055/s-0041-1730430. 34100274 10.1055/s-0041-1730430

[64] Cavolo A, De Casterlé BD, Naulaers G, Gastmans C. Neonatologists’ decision-making for resuscitation and non-resuscitation of extremely preterm infants: ethical principles, challenges, and strategies. BMC Med Ethics. 2021;22(1):129. 10.1186/s12910-021-00702-7. 34563198 10.1186/s12910-021-00702-7 PMC8467007

[65] Silberberg A, Villar MJ, Torres S. Opinions of Argentinean neonatologists on the initiation of life-sustaining treatment in preterm infants. Health Sci Rep. 2018;1(12):e100. 10.1002/hsr2.100. 30623054 10.1002/hsr2.100 PMC6295614

[66] Tucker Edmonds B, McKenzie F, Panoch JE, Wocial LD, Barnato AE, Frankel RM. Doctor, what would you do? Physicians’ responses to patient inquiries about periviable delivery. Patient Educ Couns. 2015;98(1):49–54. 10.1016/j.pec.2014.09.014. 25373527 10.1016/j.pec.2014.09.014 PMC4250443

[67] Arzuaga B, Adam H, Ahmad M, Padela A. Attitudes toward the resuscitation of periviable infants: a national survey of American Muslim physicians. Acta Paediatr. 2016;105(3):260–7. 10.1111/apa.13222. 26399580 10.1111/apa.13222

[68] Campo-Engelstein L, Andaya E. Clinicians’ criteria for fetal moral status: viability and relationality, not sentience. J Med Ethics. 2024;50(9):634–9. 10.1136/jme-2022-108392. 36347605 10.1136/jme-2022-108392

[69] Wang D, Li L, Ming BW, Ou CQ, Han T, Cao J, et al. Differences in the attitudes toward resuscitation of extremely premature infants between neonatologists and obstetricians: a survey study in China. Front Pediatr. 2023;11:1308770. 10.3389/fped.2023.1308770. 38152648 10.3389/fped.2023.1308770 PMC10751309

[70] Curkovic M, Rubic F, Jozepovic A, Novak M, Filipovic-Grcic B, Mestrovic J, et al. Navigitating the shadows: medical professionals’ values and perspectives on end-of-life care within pediatric intensive care units in Croatia. Front Pediatr. 2024;12:1394071. 10.3389/fped.2024.1394071. 39188642 10.3389/fped.2024.1394071 PMC11345198

[71] Arbour K, Lindsay E, Laventhal N, Myers P, Andrews B, Klar A, Dunbar AE 3rd. Shifting provider attitudes and institutional resources surrounding resuscitation at the limit of gestational viability. Am J Perinatol. 2022;39(8):869–77. 10.1055/s-0040-1719. 33111279 10.1055/s-0040-1719071

[72] Krick JA, Feltman DM. Neonatologists’ preferences regarding guidelines for periviable deliveries: do we really know what we want? J Perinatol. 2019;39:445–52. 10.1038/s41372-019-0313-1. 30659238 10.1038/s41372-019-0313-1

[73] American Society for Bioethics and Humanities. Core competencies for healthcare ethics consultation. 3rd ed. Glenview (IL): American Society for Bioethics and Humanities; 2025. https://asbh.org/resources/books.

[74] Todini C, Corsano B, Giardina S, Masilla SS, Raimondi C, Refolo P, et al. Ethical decision-making and clinical ethics support in Italian neonatal intensive care units: Results from a national survey. Healthc (Basel). 2022;10(12):2459. 10.3390/healthcare10122459. 10.3390/healthcare14020181 PMC12841293 41595317

[75] Gasparetto A, Jox RJ, Picozzi M. The notion of neutrality in clinical ethics consultation. Philos Ethics Humanit Med. 2018;13:3. 10.1186/s13010-018-0056-1. 29482585 10.1186/s13010-018-0056-1 PMC5828077

[76] Gasparetto A, Pegoraro R, Picozzi M. Goals change roles: how does the clinic redefine philosophical critical distance? Am J Bioeth. 2018;18(6):64–6. 10.1080/15265161.2018.1459943. 10.1080/15265161.2018.1459943 29852120

[77] Corsano B, Masilla SS, Vento G, Papacci P, Costa S, Mallardi M, et al. The experience of clinical ethics consultation at the Neonatal Intensive Care Unit of the Fondazione Policlinico Universitario A. Gemelli. IRCCS Med e Morale. 2023;72(3):329–39. 10.4081/mem.2023.1246.

[78] Göbert P, Blanckenburg PV, Maier RF, Seifart C. Utilization and evaluation of ethics consultation services in neonatal intensive care. Child (Basel). 2024;11(11):1349. 10.3390/children11111349. 10.3390/children11111349 PMC11593247 39594924

[79] Lantos JD. Ethical problems in decision making in the neonatal ICU. N Engl J Med. 2018;379(19):1851–60. 10.1056/NEJMra1801063. 30403936 10.1056/NEJMra1801063

