# Supplementary material for: Ethical challenges and moral conflicts in periviability prenatal counseling: a scoping review of healthcare professionals’ perspectives
Source: BMC Med Ethics. 2026 May 12;27:132. doi: 10.1186/s12910-026-01464-w (PMC13366718; doi:10.1186/s12910-026-01464-w)
Supplement: Supplementary file 1 — Supplementary Material 1: Supplementary File S1. Full search strategies for all databases. [file 12910_2026_1464_MOESM1_ESM.pdf]

Search strategies for all electronic databases included in the scoping review

Related article: Ethical challenges and moral conflicts in periviability prenatal counseling: a scoping review of healthcare professionals' perspectives
Last search date: July 2025

Database	Coverage years	Language	Search strategy
**PubMed / MEDLINE**	2015–2025	English	(("Perinatal Care"[Mesh] OR "Fetal Viability"[Mesh] OR periviable[tiab] OR "peri-viability"[tiab] OR periviability[tiab] OR "limit of viability"[tiab] OR "fetal viability"[tiab] OR "periviable birth"[tiab] OR "peri-viability birth"[tiab] OR "peri-viable birth"[tiab])<br>AND<br>("Bioethical Issues"[Mesh] OR "Ethics"[Mesh] OR "Ethics Consultation"[Mesh] OR bioethics[tiab] OR "ethical issues"[tiab] OR ethics[tiab] OR "ethical dilemma"[tiab] OR "ethical concerns"[tiab])<br>AND<br>("Health Personnel"[Mesh] OR "Health Occupations"[Mesh] OR healthcare workers[tiab] OR healthcare professionals[tiab] OR healthcare providers[tiab] OR healthcare personnel[tiab] OR doctor*[tiab] OR nurse*[tiab] OR obstetric*[tiab] OR neonatol*[tiab] OR gynecol*[tiab]))
**CINAHL (EBSCOhost)**	2015–2025	English	((MH "Fetal Viability") OR (MH "Perinatal Care") OR periviable OR "limit of viability" OR "periviable birth" OR "peri-viable birth" OR periviability OR "peri-viability")<br>AND<br>((MH "Ethics, Clinical") OR (MH "Ethics Consultation") OR (MH "Bioethics") OR bioethics OR ethics OR "ethical issues" OR "ethical concerns" OR "ethical dilemma")<br>AND<br>((MH "Health Personnel") OR (MH "Physicians") OR (MH "Nurses") OR (MH "Midwives") OR (MH "Neonatology") OR "healthcare provider*" OR "healthcare professional*" OR doctor* OR nurse* OR obstetric* OR neonatol* OR gynecol*)
**APA PsycINFO (EBSCOhost)**	2015–2025	English	((MH "Fetal Viability") OR (MH "Perinatal Care") OR periviable OR "peri-viability" OR periviability OR "limit of viability" OR "fetal viability" OR "periviable birth" OR "peri-viable birth")<br>AND<br>((MH "Ethics") OR (MH "Bioethics") OR (MH "Ethical Issues") OR (MH "Ethical Decision Making") OR (MH "Ethics Consultation") OR bioethics OR "ethical issues" OR ethics OR "ethical concerns" OR "ethical dilemma")<br>AND<br>((MH "Health Personnel") OR (MH "Health Care Providers") OR healthcare worker* OR healthcare professional* OR healthcare provider* OR doctor* OR physician* OR nurse* OR obstetric* OR neonatol* OR gynecol*)
**Web of Science – Core Collection**	2015–2025	English	TS=(periviable OR limit of viability OR periviability OR periviable birth OR peri-viable birth)<br>AND TS=(bioethics OR ethics OR ethical issues OR ethical dilemma OR ethical concerns OR ethics consultation)<br>AND TS=(healthcare provider* OR healthcare professional* OR healthcare personnel OR healthcare worker* OR doctor* OR nurse* OR obstetric* OR neonatol* OR gynecol*)
**Psychology and Behavioral Sciences Collection (EBSCOhost)**	2015–2025	English	(TX(periviable) OR TX("peri-viability") OR TX(periviability) OR TX("limit of viability") OR TX("fetal viability") OR TX("periviable birth") OR TX("peri-viable birth"))<br>AND<br>(TX(bioethics) OR TX("ethical issues") OR TX(ethics) OR TX("ethical concerns") OR TX("ethical dilemma") OR TX("ethics consultation"))<br>AND<br>(TX("healthcare worker*") OR TX("healthcare professional*") OR TX("healthcare provider*") OR TX(doctor*) OR TX(physician*) OR TX(nurse*) OR TX(obstetric*) OR TX(neonatol*) OR TX(gynecol*))

All database-specific search strategies were adapted using controlled vocabulary where available (e.g., MeSH, CINAHL Headings, APA Thesaurus) and equivalent free-text terms. Search strategies were reviewed and validated in collaboration with a health sciences librarian.
